# Role of iron, zinc and reduced glutathione in oxidative stress induction by low pH in rat brain synaptosomes

**DOI:** 10.1186/2193-1801-3-560

**Published:** 2014-09-26

**Authors:** Tatyana G Pekun, Sviatlana V Hrynevich, Tatyana V Waseem, Sergei V Fedorovich

**Affiliations:** Laboratory of Biophysics and Engineering of Cell, Institute of Biophysics and Cell Engineering, Akademicheskaya St., 27, Minsk, 220072 Belarus; INSERM UMR1106, Institut de Neurosciences des Systems, Aix-Marseille University, Marseille, France

**Keywords:** Synaptosomes, Acidosis, ischemia, Iron, Zinc, Glutathione

## Abstract

Brain ischemia leads to a decrease in pH_o_. We have shown previously in synaptosomes that the extracellular acidification induces depolarization of mitochondria followed by synthesis of superoxide anions and oxidative stress. Here, we investigated the effects of lowered pH_o_ on oxidative stress and membrane potentials in synaptosomes treated by the iron chelator deferoxamine and zinc chelator TPEN. We demonstrated that chelating of metals has no impact on superoxide anion synthesis and intrasynaptosomal mitochondria depolarization. Meanwhile, deferoxamine was able to inhibit oxidative stress induced by low pH_o_ and hydrogen peroxide application. Compared to deferoxamine, TPEN was less effective but it decreased the DCF fluorescence induced by pH_o_ 6.0 which had no effects in other oxidative stress models. We found that the chelators were able to inhibit slightly plasma membrane depolarization. Synaptosomes preincubation at low pH_o_ caused no effects on the reduced glutathione level. Depletion of glutathione by CDNB produced no additional increase in the DCF fluorescence induced by pH_o_ 7.0. Our results suggest that free iron is crucial for the development of oxidative stress elicited by acidification in synaptosomes. Chelating of this metal seems to be a promising strategy for protecting the neuronal presynaptic terminals against oxidative stress developed at stroke.

## Introduction

Stroke is associated with acidification reaching pH_o_ of 5.3 in certain cases, for instance, in hyperglycemia (Thorn and Heitmann 1954; Crowell and Kaufman, 1961; Kraig and Chesler 1990; Tombaugh and Sapolsky 1993; Isaev et al. 2008). The main cause of pH lowering is a metabolic shift to predominance of glycolysis (Tombaugh and Sapolsky 1993; Isaev et al. 2008; Obara et al. 2008). Apart form ischemia, acidification was also observed in several neurodegenerative diseases (Yates et al. 1990) potentially contributing to their pathogenesis.

Lowering of pH down to 6.0 can induce neuronal death (Nedergaard et al. 1991; Isaev et al. 2010). The main cause of acid-induced neuronal death is thought to be activation of the acid sensitive ion channels (ASICs) (Krishtal and Pidoplichko 1981; Xiong et al. 2004; Isaev et al. 2008; Wemmie et al. 2013). However, at least in some cases, damage of neurons under low pH was associated rather with an acidification-induced increase in cytosolic zinc levels than ASIC activity (Isaev et al. 2010; Kiedrowski 2011). It was suggested that the mitochondria depolarization followed by oxidative stress plays a key role in development of this phenomenon (Isaev et al. 2010).

It was shown that lowering of pH may lead to an increase of free radical formation in the brain homogenates, slices and isolated neuronal presynaptic terminals termed synaptosomes (Siesjo et al. 1985; Bralet et al. 1991, 1992; Pekun et al. 2012, 2013).

Recently, we have demonstrated that superoxide anion synthesis in mitochondria followed by their depolarization is the primary cause of oxidative stress induced by extracellular acidification (Pekun et al. 2013). Nonetheless, the release of iron from proteins and inhibition of enzymes maintaining the cellular pool of reduced glutathione were reported to exert the crucial effects on development of oxidative stress in brain homogenates, brain slices, and neurons (Siesjo et al. 1985; Bralet et al. 1992; Ying et al. 1999; Lewerenz et al. 2010). It is unknown whether iron, zinc and glutathione have any role in the development of oxidative stress in synaptosomes, an experimental model that we have characterized earlier (Pekun et al. 2012, 2013). Meanwhile, it was demonstrated that the local free radical formation in synapses is able to modify significantly the synaptic vesicle recycling (Giniatullin et al. 2006; Keating 2008; Tarasenko et al. 2012; Tsentsevitsky et al. 2013). Accordingly, synaptic oxidative stress induced by low pH_o_ might underlay irreversible impairment of synaptic transmission which is a poorly investigated consequence of brain ischemia (Hofmeijer and van Putten 2012).

In the present, paper we investigated an impact of the membrane permeable iron chelator deferoxamine and membrane permeable zinc chelator N,N,N’,N’-tetrakis(2-pyridylmethyl)ethylenediamine (TPEN) on free radical formation in rat brain synaptosomes at low pH_o_. Reactive oxygen species (ROS) accumulation was monitored by the fluorescent dye DCFDA and dihydroethidium (LeBel and Bondy 1990; Pekun et al. 2013). It is well known that depolarization of mitochondrial membrane can cause free radical formation (Votyakova and Reynolds 2001; Abramov et al. 2007; Manzanero et al. 2013; Pekun et al. 2013) and a subsequent ROS accumulation is able to result in depolarization of the neuronal plasma membrane (Bao et al. 2005; Nani et al. 2010). Acidification decreases potentials in either mitochondrial or plasma membrane of rat brain synaptosomes (Fedorovich et al. 1996, 2003; Pekun et al. 2013, 2014); therefore, we investigated the effects of chelators on mitochondrial or plasma membrane potentials. Plasma membrane potential was monitored by a fluorescent dye DiSC3(5) (Waseem and Fedorovich 2010), mitochondrial potential was monitored by a fluorescent dye JC-1 (Chinopoulos et al. 1999). Further, we investigated the intrasynaptosomal concentration of reduced glutathione after lowering of pHo. Glutathione was monitored by a fluorescent dye monochlorobimane (Kamencic et al. 2000; Abramov et al. 2007).

## Materials and methods

### Materials

Dihydroethidium, 2’,7’-dichlorodihydrofluorescein diacetate (DCFDA), oligomycin, 3,3’ – dipropylthiadicarbocyanine (DiSC3(5)), deferoxamine mesylate, butylated hydroxytoluene (ionol), 1-chloro-2,4-dinitrobenzene (CDNB), monochlorobimane and N, N, N’, N’-tetrakis(2-pyridylmethyl)ethylenediamine (TPEN) were purchased from Sigma (St. Louis, MO, USA). 4-(2-Hydroxyethyl)piperazine-N’-1-ethanesulfonic acid (HEPES) was obtained from Merck (Darmstadt, Germany). 5,5’,6,6’-tetrachloro-1,1’,3,3’-tetraethylbenzimidazolo-carbocyanine iodide (JC-1) and rotenone were received from Calbiochem (La Jolla, CA, USA). 4-morpholineethanesulfonic acid (MES) was purchased from Reanal (Budapest, Hungary). Tris(hydroxymethyl)aminomethane (Tris) was obtained from BDH (Poole, UK).

### Synaptosomes preparation

Synaptosomes were isolated from brain hemispheres of 12-16-week-old male Wistar rats according to Hajos ( 1975). Stock suspensions of synaptosomes (10 mg/ml) were prepared in medium A (composition in mM: 132 NaCl, 5 KCl, 10 glucose, 1.3 MgCl_2_, 1.2 NaH_2_PO_4_, 15 HEPES, 5 Tris, pH 7.4, 310 mOsm/l) and kept on ice. Animal experiments were carried out in accordance with EU Directive 2010/63/EU.

### Intrasynaptosomal ROS determination

Intrasynaptosomal ROS was monitored by fluorescent dye DCFDA according to LeBel and Bondy ( 1990) with modifications according to Alekseenko et al. ( 2008).

Synaptosomes purification was carried out in medium A and then after additional washing the pellet was resuspended in the same medium (protein concentration 10 mg/ml). Suspension was incubated for 60 min at 37°C in presence of 25 μM DCFDA. Extracellular dye was removed by sedimentation and the final pellet was resuspended in 2 ml calcium-free medium B (composition in mM: 132 NaCl, 5 KCl, 10 glucose, 1.3 MgCl_2_, 1.2 NaH_2_PO_4_, 2.0 CaCl_2_, 10 HEPES, 10 MES, pH 6.0-7.4, 310 mOsm/l). To investigate ROS formation, 200 μl of loaded synaptosomes were added to the cuvette containing 1.8 ml of incubation medium B Fluorescence intensity was recorded at λ_ex/em_ = 501/525 nm on spectrofluorimeter Cary Eclipse (“Varian”, USA) with constant stirring and 37°C temperature.

To change the extracellular pH, the aliquots of 60 μl of HCl solution having different acid concentrations were directly added to the cuvette at 50 s. The same quantity of water was added in control experiments. The control curve was extracted from the experimental curve.

### Determination of superoxide anion formation

Superoxide anion level was determined by fluorescent dye dihydroethidium according to Pekun et al. ( 2013).

Synaptosomes purification was carried out in calcium-free medium A. Synaptosomal pellet was resuspended in calcium-free medium B. An aliquot of synaptosome suspension (200 μl) was added to the cuvette containing 1.8 ml of incubation medium B with 2.0 mM CaCl_2_. 5 μM of dihydroethidium were added to the cuvette, then after 1 minute different additions were made. Fluorescence intensity was recorded at λ_ex/em_ = 490/560 nm on spectrofluorimeter Cary Eclipse (“Varian”, USA) at constant stirring and 37°C.

To change the extracellular pH the aliquots of 60 μl of HCl solution having different acid concentrations were added to the cuvette directly. The same quantity of water was added in control experiments. The control curve was extracted from the experimental curve.

### Determination of intrasynaptosomal mitochondria membrane potential by fluorescent dye JC-1

Membrane potential of intrasynaptosomal mitochondria was detected by fluorescent dye JC-1 according to Chinopoulos et al. ( 1999) with modifications according to Pekun et al. ( 2013).

Synaptosomes purification was carried out in calcium-free medium A and then the pellet was resuspended in the same medium (protein concentration of 5 mg/ml). Suspension was incubated for 15 min at 37°C in the presence of 10 μg/ml dye. Extracellular dye was washed out three times by sedimentation and the final pellet was resuspended in 2.0 ml calcium-free medium B (protein concentration of 10 mg/ml).

To investigate mitochondrial membrane potential, 200 μl of loaded synaptosomes were added to the cuvette containing 1.8 ml of incubation medium B. Fluorescence intensity was recorded at λ_ex/em_ = 504/535 nm on spectrofluorimeter Cary Eclipse (“Varian”, USA) at constant stirring and 37°C.

To change the extracellular pH, 60 μl of HCl solutions having different acid concentrations were directly added to the cuvette on 50s. The same quantity of water was added in control experiments. The control curve was extracted from the experimental curve.

### Investigation of plasma membrane potential

Plasma membrane potential was investigated using fluorescent dye 3,3’–dipropylthiadicarbocianyne (DiSC3(5)) according to Waseem and Fedorovich ( 2010). An aliquot of synaptosome suspension (200 μl) was added to the cuvette containing 2 ml of incubation medium B. After 1 min 1 μM of DiSC3(5) was added to the cuvette. After 1 minute, 10 μM of rotenone and 5 μg/ml oligomycin was added. Fluorescence intensity was recorded at λ_ex/em_ = 640/688 nm on spectrofluorimeter Cary Eclipse (“Varian”, USA) at constant stirring and 37°C. Synaptosomes were preincubated with different chelators for 30 min at 37°C. All indicated compounds also were present in incubation medium throughout the fluorescence measurements.

To change the extracellular pH, 60 μl of HCl solutions having different acid concentrations were directly added to the cuvette in 1 minute after addition of rotenone and oligomycin. The same quantity of water was added in control experiments. The control curve was extracted from the experimental curve.

### Determination of reduced glutathione

Level of reduced glutathione was estimated by fluorescent dye monochlorobimane (Kamencic et al. 2000; Abramov et al. 2007). Synaptosomes purification was carried out in calcium-free medium A. Synaptosomal pellet was resuspended in calcium-free medium B. An aliquot of synaptosomal suspension (200 μl) was added to 800 μl incubation medium B having pH 7.4, 7.0, 6.0 or containing 50 μM of 1-chloro-2,4-dinitrobenzene (CDNB). Synaptosomes were sedimented by centrifugation after 10 min incubation at 37°C. Then pellets were resuspended in 1 ml of incubation medium A containing 50 μM of monochlorobimane. Samples were incubated for 40 min at room temperature. The reaction was stopped by transferring samples on ice followed by fast centrifugation. The resulting pellets was resuspended again in 2 ml of incubation medium A, and fluorescence was measured on spectrofluorimeter Cary Eclipse (“Varian”, USA) at λ_ex/em_ = 383/485 nm.

### Other methods

Protein concentration was assayed according to (Lowry et al. 1951) using bovine serum albumin as a standard. Data are presented as mean ± S.E.M. where indicated, statistical significance was evaluated using one-tailed Student’s t-test.

## Results

### Role of iron and zinc in development of oxidative stress

**Figure 1:**
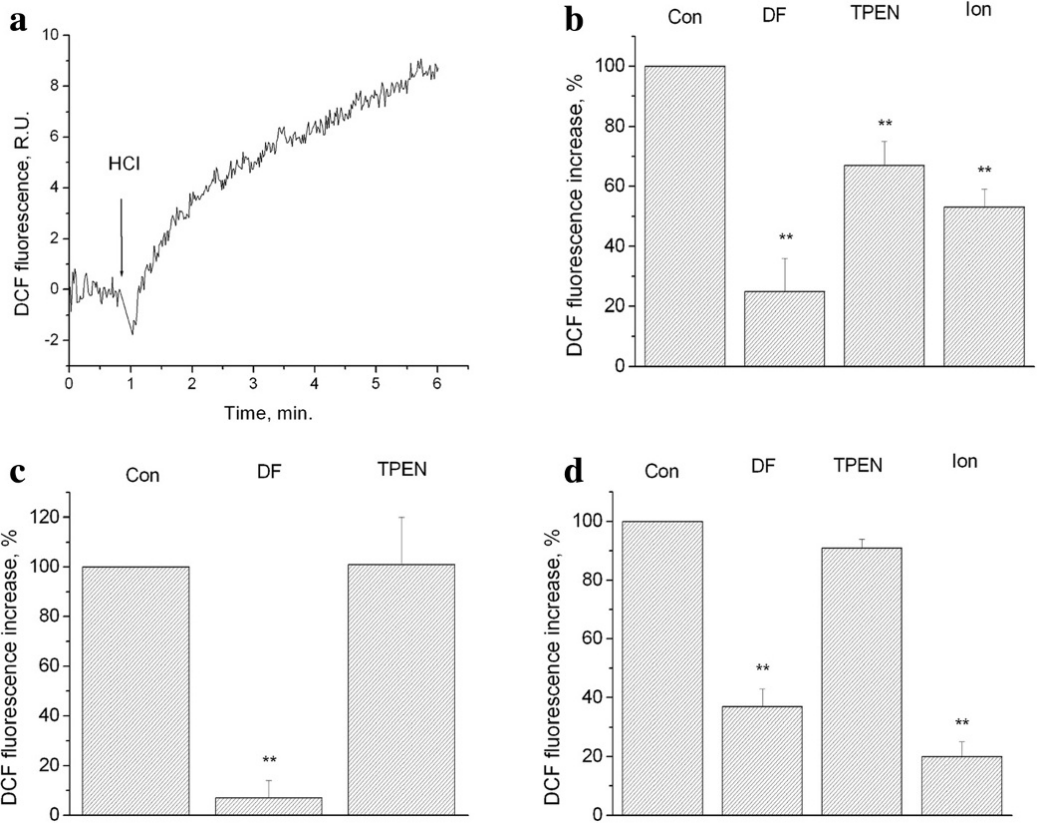
**Influence of extracellular pH on DCF fluorescence in synaptosomes.** Role of iron and zinc. **a)** Kinetics of DCF fluorescence increase after extracellular acidification. HCl down to pH 6.0 was added where indicated. Curves represent 5 independent experiments. **b)** Influence of deferoxamine, TPEN and ionol on DCF fluorescence evoked by pH 6.0 **c)** Influence of desferoxamine and TPEN on DCF fluorescence evoked by pH 7.0 **d)** Influence of desferoxamine, TPEN and ionol on DCF fluorescence evoked by 1 mM H_2_O_2_. Con − control DF − synaptosomes were preincubated for 60 min at 37°C with 100 μM of deferoxamine, incubation medium contains also 100 μM of desferoxamine. TPEN - synaptosomes were preincubated for 60 min at 37°C with 5 μM of TPEN, incubation medium contains also 5 μM of TPEN Ion - synaptosomes were preincubated for 60 min at 37°C with 200 μM of ionol, incubation medium contains also 200 μM of ionol. Bars represent DCF fluorescence increase within 4 minutes after additions. Data presented are mean values±SEM of at least 4 experiments. 100% level corresponds to fluorescence increase in response to pH 6.0 (b), pH 7.0 (c), 1 mM of H_2_O_2_ (d) without chelators and antioxidants (b), **P≤0.01 vs. 100%.

Figure 1a illustrates that decreasing of pH_o_ down to 6.0 results in an increase in DCF fluorescence, as we had demonstrated earlier (Pekun et al. 2013). As expected, this pH_o-_induced increase was sensitive to the antioxidant ionol (200 μM) (Figure 1b). In these experimental conditions, oxidative stress was inhibited by the iron chelator deferoxamine (100 μM) and zinc chelator TPEN (5 μM), with deferoxamine being even more effective than ionol (Figure 1b). Conversely, oxidative stress induced by a moderate acidification (pH_o_ 7.0) was sensitive to deferoxamine, but not to TPEN (Figure 1c). The increase in DCF fluorescence induced by 1 mM H_2_O_2_ was sensitive to ionol and deferoxamin, but not to TPEN (Figure 1d).

### Role of iron and zinc in superoxide anion synthesis

**Figure 2:**
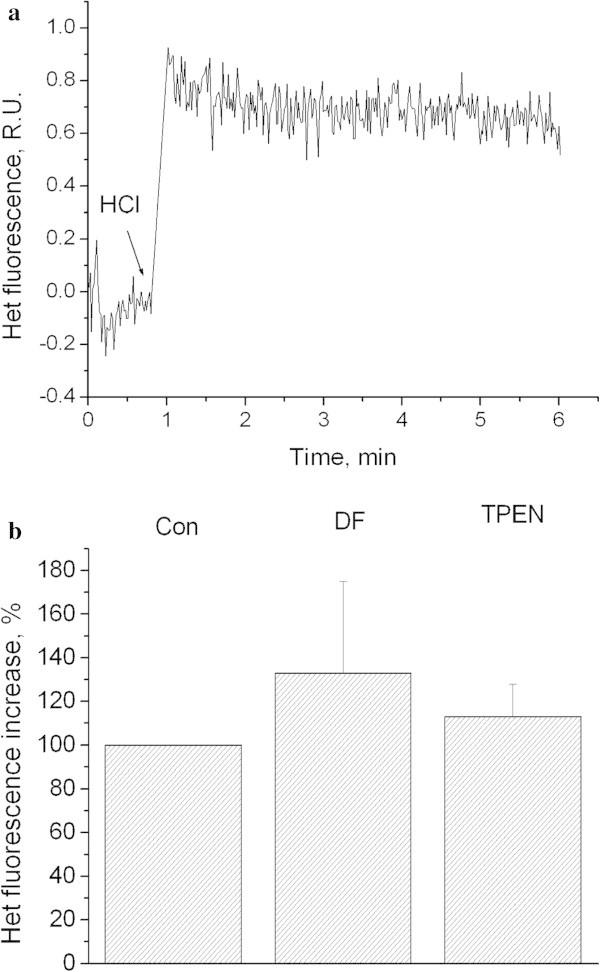
**Influence of low pH on dihydroethidium fluorescence in synaptosomes.** Role of iron and zinc. **a)** Kinetics of dihydroethidium fluorescence increase after extracellular acidification. HCl down to pH 6.0 was added where indicated. Curves represent 22 independent experiments. **b)** Influence of desferoxamine and TPEN on DCF fluorescence evoked by pH 6.0. Con − control. DF − synaptosomes were preincubated for 30 min at 37°C with 100 μM of deferoxamine, incubation medium contains also 100 μM of desferoxamine. TPEN - synaptosomes were preincubated for 30 min at 37°C with 5 μM of TPEN, incubation medium contains also 5 μM of TPEN. Bars represent dihydroethidium fluorescence increase within 4 minutes after additions. Data presented are mean values ± SEM of at least 4 experiments. 100% level corresponds to fluorescence increase in response to pH 6.0 without chelators and antioxidants.

Figure 2a shows that decreasing of pH_o_ down to 6.0 results in the elevation dihydroethidium fluorescence, as we had demonstrated earlier (Pekun et al. 2013). This effect was not abolished by application of iron and zinc chelators (Figure 2b).

### Role of iron and zinc in induction of intrasynaptosomal mitochondria depolarization

**Figure 3:**
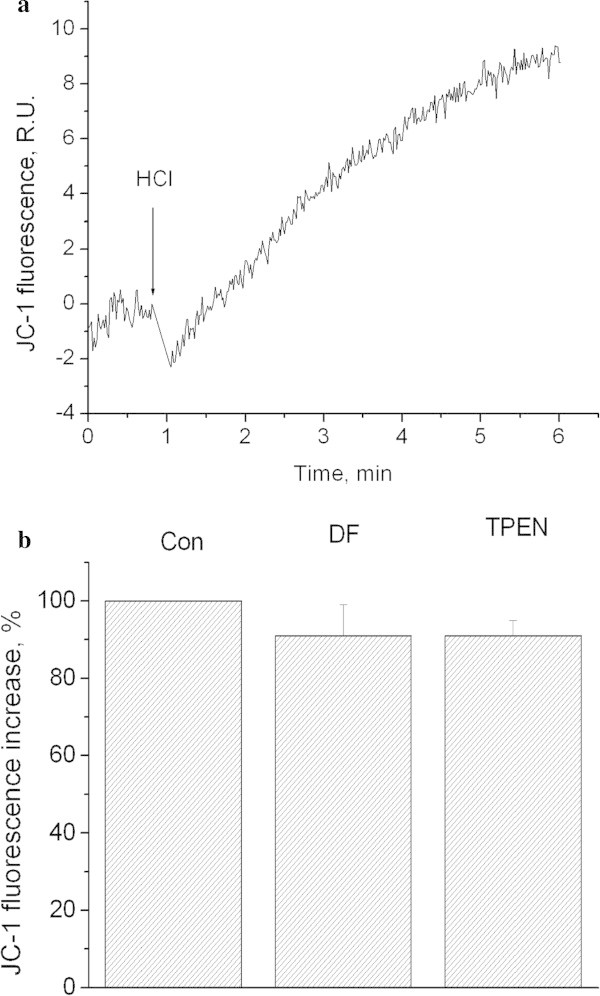
**Influence of extracellular pH on JC-1 fluorescence.** Role of iron and zinc. **a)** Kinetics of JC-1 fluorescence increase after extracellular acidification. HCl down to 6.0 was added where indicated. Curves represent 7 independent experiments. **b)** Influence of deferoxamine and TPEN on JC-1 fluorescence evoked by pH 6.0. Con − control. DF − synaptosomes were preincubated for 30 min at 37°C with 100 μM of deferoxamine, incubation medium contains also 100 μM of deferoxamine. TPEN - synaptosomes were preincubated for 30 min at 37°C with 5 μM of TPEN, incubation medium contains also 5 μM of TPEN. Bars represent JC-1 fluorescence increase within 4 minutes after additions. Data presented are mean values ± SEM of at least 4 experiments. 100% level corresponds to fluorescence increase in response to pH 6.0.

Figure 3a shows that decreasing of pH_o_ down to 6.0 results in the elevation JC-1 fluorescence, as we had demonstrated earlier (Pekun et al. 2013). This effect was not abolished by application of iron and zinc chelators (Figure 3b).

### Role of iron and zinc in induction of plasma membrane depolarization

**Figure 4:**
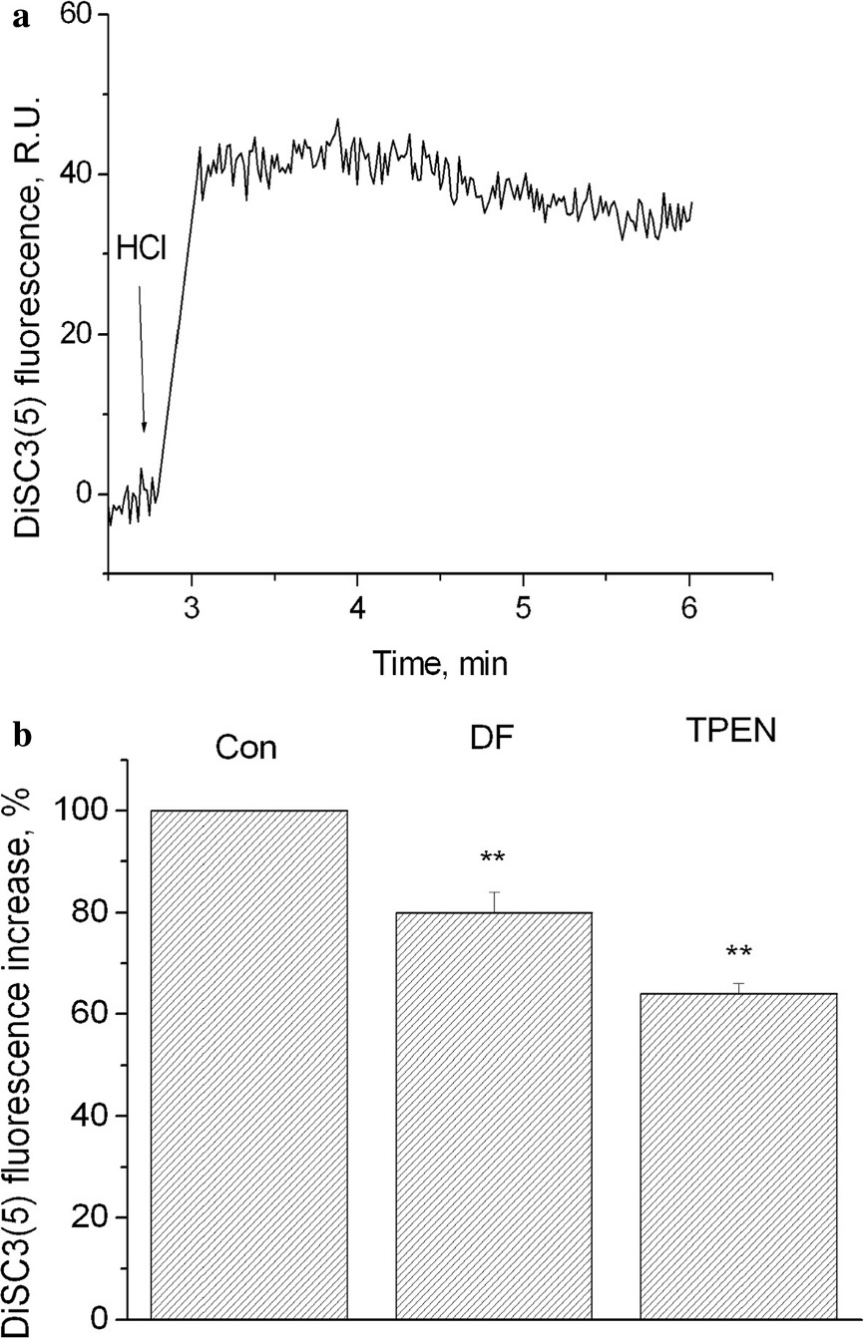
**Influence of extracellular pH on DiSC3(5) fluorescence.** Role of iron and zinc. **a)** Kinetics of DiSC3(5) fluorescence increase after extracellular acidification. HCl down to 6.0 was added where indicated. Curves represents 4 independent experiments. **b)** Influence of desferoxamine and TPEN on DiSC3(5) fluorescence evoked by pH 6.0. Con − control. DF − synaptosomes were preincubated for 30 min at 37°C with 100 μM of deferoxamine, incubation medium contains also 100 μM of deferoxamine. TPEN - synaptosomes were preincubated for 30 min at 37°C with 5 μM of TPEN, incubation medium contains also 5 μM of TPEN. Bars represent DiSC3(5) fluorescence increase within 3 minutes after additions. Data presented are mean values ± SEM of at least 4 experiments. 100% level corresponds to fluorescence increase in response to pH 6.0. **P ≤ 0.01 vs. 100%.

Figure 4a shows that decreasing of pH_o_ down to 6.0 results in the elevation DiSC3(5) fluorescence, as we had demonstrated earlier (Pekun et al. 2014). This effect was sensitive to iron and zinc chelators (Figure 4b). Furthermore, TPEN was more effective than deferoxamine (Figure 4b).

### Role of reduced glutathione in induction of oxidative stress

**Figure 5:**
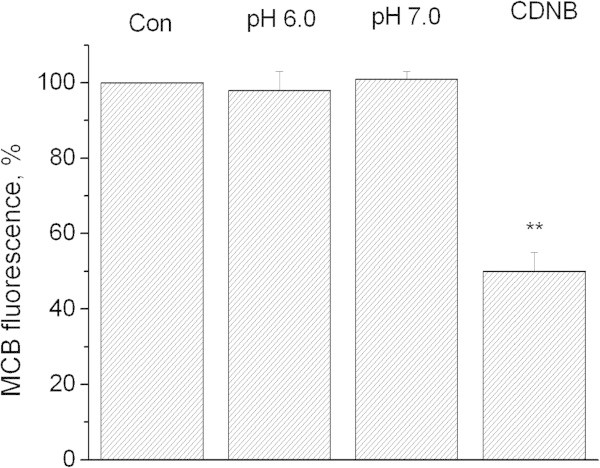
**Influence of preincubation at low pH on fluorescence of complex glutathione-monochlorobimane.** Con − control. pH 6.0 – synaptosomes were preincubated for 10 min at pH 6.0. pH 7.0 - synaptosomes were preincubated for 10 min at pH 7.0. CDNB – synaptosomes were preincubated for 10 min with 50 μM of CDNB. Data presented are mean values ± SEM of at least 4 experiments. 100% level corresponds to fluorescence in control. **P ≤ 0.01 vs. 100%.

**Figure 6:**
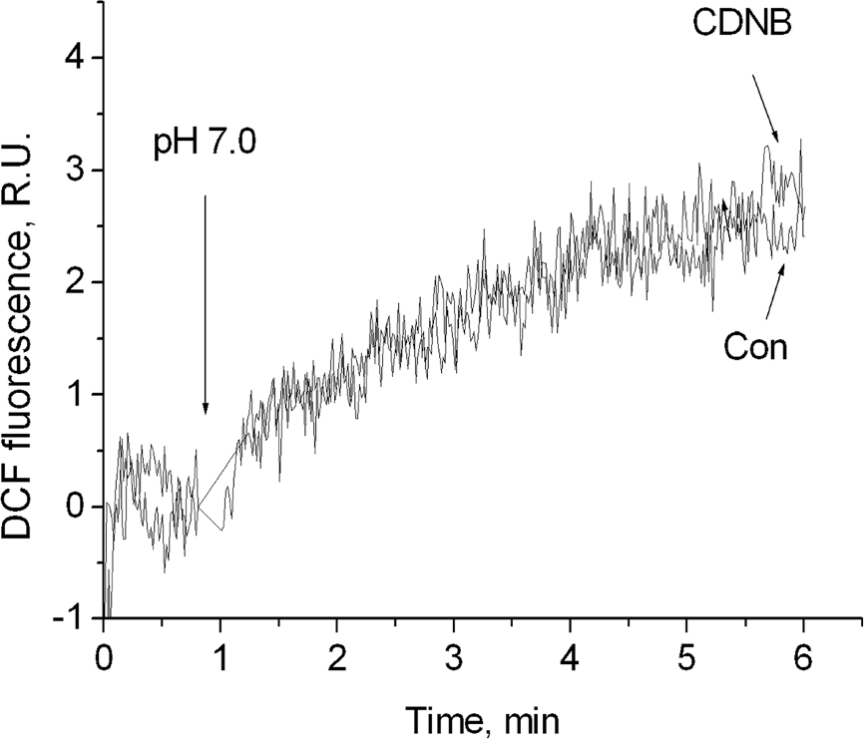
**Influence of gluthatione depletion on DCF fluorescence increase evoked by pH 7.0.** HCl down to pH 7.0 was added where indicated. Con- control. CDNB - synaptosomes were preincubated for 10 min with 50 μM of CDNB.

Figure 5 shows that incubation of synaptosomes at pH_o_ 6.0 for 10 minutes does not change the levels of reduced glutathione. Conversely, treatment with 1-chloro-2,4-dinitrobenzene (CDNB) of the same duration decreased the monochlorobimane fluorescence indicating glutathione depletion (Figure 5). Furthermore, we have shown that the pattern of oxidative stress development detected by DCF upon lowering of pH_o_ to 7.0 is similar between control synaptosomes and synaptosomes wherein the pool of reduced glutathione has been depleted by CDNB (Figure 6).

## Discussion

In order to investigate the process of free radicals accumulation we have used the fluorescent dyes DCFDA and dihydroethidium. DCFDA indicates predominantly levels of highly toxic OH radicals, while dihydroethidium is able to detect superoxide anion which can function as a signaling molecule apart from its damaging effects. This signaling function may be important for protecting the brain against ischemia (LeBel and Bondy 1990; Halliwell 2006; Ravati et al., 2001; D’Autreaux and Toledano 2007; Niizuma et al. 2009; Kalyanaraman et al. 2012).

We have shown that iron chelator is able to block the · OH radical formation and plasma membrane depolarization, but has no effects on the superoxide anion synthesis and mitochondria depolarization (Figures 1, 2, 3 and 4). Therefore, the presence of free iron is considered to be an essential prerequisite contributing to the damage of presynaptic terminals upon lowering pH. Furthermore, we have shown that the development of significant oxidative stress can potentially be obviated through the use of iron chelators, even when the superoxide anion synthesis is increased. The presence of iron is also important for the hydroxyl radical formation induced by hydrogen peroxide (Figure 1d). Deferoxamine is able to inhibit oxidative stress induced by both strong and moderate acidification (Figure 1b, c).

The effect of deferoxamine, in terms of smaller oxidative stress induced by hydrogen peroxide, was comparable with that of the classical lipophilic antioxidant ionol (Hocman 1988) (Figure 1d), although the effect was even stronger in the extracellular acidification model (Figure 1b).

Two important conclusions can be drawn based on our experiments aimed at investigating the effect of deferoxamine on plasma membrane and intrasynaptosomal mitochondria potentials.

1. Iron chelating inhibited the plasma membrane depolarization, but not the mitochondria depolarization (Figures  3 and 4). This confirmed our previous findings indicating different mechanisms of acidosis-induced reduction of plasma membrane and mitochondria potentials (Pekun et al. 2014).
2. Our results suggest that ROS are involved in the depolarization of synaptosomal plasma membrane. We have demonstrated previously that the decrease of potential in such case was induced by the inhibition of sodium pump and potassium channels (Fedorovich et al. 2003). Therefore, the free radical-induced damage of sodium pump and/or potassium channels in association with direct influence of protons on potassium channels may underlay the synaptosomal plasma membrane depolarization (Moody 1984).

Our results with chelator TPEN (Figures 2 and 3) rule out the leading role of zinc in mitochondria depolarization and superoxide anion synthesis, as it was shown for other cell models of stroke (Medvedeva et al. 2009; Sensi et al. 2009). However, we show that TPEN is able to inhibit oxidative stress induced by strong but not moderate acidification (Figure 1b, c). Its effect was less pronounced as compared to the effect of deferoxamine (Figure 1b). In this case, the antioxidant properties of TPEN are thought to result from chelating of copper or even chelating of iron with low affinity (Ying et al. 1999; Armstrong et al. 2001; Medvedeva et al. 2009) rather than binding of zinc. Although we used a very low TPEN concentration (5 μM), we could not exclude a possibility of the involvement of other prooxidant metals.

We have shown that TPEN was able to inhibit the plasma membrane depolarization more strongly than deferoxamine (Figure 4b). This suggests that zinc is more likely involved in the acid-induced decrease of plasma membrane potential than in the decrease of mitochondria potential.

Brain ischemia was shown to change significantly the intracellular levels of reduced glutathione. These levels appeared to be low in ischemic core and surprisingly high in penumbra (Bragin et al. 2010). Furthermore, acidification is found to induce glutathione depletion in neurons (Lewerenz et al. 2010). In contrast, our results clearly show that the levels of reduced glutathione do not change in experimental models used in our studies (Figure 5). In addition, depletion of glutathione by CDNB does not intensify the oxidative stress induced by moderate acidification (Figure 6). Therefore, our results suggest that the reduced glutathione does not contribute significantly to the antioxidant protection of neuronal presynaptic terminals, at least in the acidosis-induced model of oxidative stress.

Deferoxamine exhibited protective effects in some experimental models of brain ischemia in vivo, for instance in ischemia associated with hyperglycemia (Xing et al. 2009) or neonatal brain ischemia (Palmer et al. 1994). It was shown that hyperglycemia could significantly intensify acidification in stroke (Kraig and Chesler 1990; Tombaugh and Sapolsky 1993). Our results provide a possible explanation of deferoxamine efficiency in this case. The clinical trials with administration of deferoxamine for treatment of hemorrhagic stroke have been recently initiated (Xi et al. 2014). Our results indicate that this compound can also be useful in protecting from damage caused by ischemic stroke especially that associated with hyperglycemia. Additionally, the synthetic chelators of iron, VK-28 and HLA-20, which display superior penetrability through blood brain barrier compared to deferoxamine (Zecca et al. 2004) and plant flavonoids, seem to be very promising compounds for the potential treatments of stroke. Flavonoids combine the properties of antioxidants and metal chelators including iron chelators (Afanas’ev et al. 1989; Mandel et al. 2008).

Our results indicate that chelating of iron seems to be a better strategy for the protection of neuronal presynaptic terminals from oxidative stress. This approach obviates the production of highly toxic hydroxyl radicals, but helps to maintain the same level of superoxide anion, which might be important for the protecting brain against ischemia.
